# Increased Anatomical Specificity of Neuromodulation via Modulated Focused Ultrasound

**DOI:** 10.1371/journal.pone.0086939

**Published:** 2014-02-04

**Authors:** Edin Mehić, Julia M. Xu, Connor J. Caler, Nathaniel K. Coulson, Chet T. Moritz, Pierre D. Mourad

**Affiliations:** 1 Department of Bioengineering, University of Washington, Seattle, Washington, United States of America; 2 Department of Materials Science and Engineering, University of Washington, Seattle, Washington, United States of America; 3 Department of Rehabilitation Medicine, University of Washington, Seattle, Washington, United States of America; 4 Department of Physiology and Biophysics, University of Washington, Seattle, Washington, United States of America; 5 Applied Physics Laboratory, University of Washington, Seattle, Washington, United States of America; 6 Department of Neurological Surgery, University of Washington, Seattle, Washington, United States of America; 7 Department of Engineering and Mathematics, University of Washington, Bothell, Washington, United States of America; University of Bologna, Italy

## Abstract

Transcranial ultrasound can alter brain function transiently and nondestructively, offering a new tool to study brain function now and inform future therapies. Previous research on neuromodulation implemented pulsed low-frequency (250–700 kHz) ultrasound with spatial peak temporal average intensities (I_SPTA_) of 0.1–10 W/cm^2^. That work used transducers that either insonified relatively large volumes of mouse brain (several mL) with relatively low-frequency ultrasound and produced bilateral motor responses, or relatively small volumes of brain (on the order of 0.06 mL) with relatively high-frequency ultrasound that produced unilateral motor responses. This study seeks to increase anatomical specificity to neuromodulation with modulated focused ultrasound (mFU). Here, ‘modulated’ means modifying a focused 2-MHz carrier signal dynamically with a 500-kHz signal as in vibro-acoustography, thereby creating a low-frequency but small volume (approximately 0.015 mL) source of neuromodulation. Application of transcranial mFU to lightly anesthetized mice produced various motor movements with high spatial selectivity (on the order of 1 mm) that scaled with the temporal average ultrasound intensity. Alone, mFU and focused ultrasound (FUS) each induced motor activity, including unilateral motions, though anatomical location and type of motion varied. Future work should include larger animal models to determine the relative efficacy of mFU versus FUS. Other studies should determine the biophysical processes through which they act. Also of interest is exploration of the potential research and clinical applications for targeted, transcranial neuromodulation created by modulated focused ultrasound, especially mFU’s ability to produce compact sources of ultrasound at the very low frequencies (10–100s of Hertz) that are commensurate with the natural frequencies of the brain.

## Introduction

Modulation of brain function via ultrasound and other applications of therapeutic ultrasound originated with the Fry brothers [1], [2]. Through excitation and inhibition of neuronal tissue, they were able to induce transient physiological effects without observable damage. Tyler and colleagues revitalized this concept [3], first showing neuron activation in a mouse brain-slice model. Next, through the use of transcranial pulsed ultrasound with a relatively large focus directed at the brains of mice, they induced observable, generally biliateral peripheral motor activity such as tail and paw flicks and whisker movements, demonstrating that ultrasonic neuromodulation (UNMOD) could stimulate entire brain circuits. These UNMOD studies [3]–[5] employed ultrasound emitted by readily available planar transducers as the source of stimulation. Even with acoustic waveguides [6], the resulting acoustic fields have lacked optimal anatomical specificity. Two recent *in-vivo* studies have used focused ultrasound (FUS), balancing relatively lower frequencies with relatively larger volumes of brain stimulation. In the first study, Yoo et al. [7] used a pulsed, 690-kHz focused ultrasound protocol on anesthetized rabbits, showing via functional magnetic resonance imaging (fMRI), electromyography (EMG), and gross observation that one side of the brain can be stimulated, inducing motor function on the contralateral side. The second study [8] used a 350-kHz focused ultrasound protocol to stimulate at least a cranial nerve associated with control of an eye of an anesthetized rat, with motion induced ipsilateral to the stimulation zone.

This study seeks to demonstrate a new method to deploy low-frequency FUS-based UNMOD, one with significantly increased anatomical specificity yet with the potential to deploy very low ultrasound frequencies through use of vibro-acoustography techniques [9], [10]. Typical research-grade embodiments of vibro-acoustography use two confocal sources of high-frequency ultrasound each run at slightly different and typical high carrier frequencies to produce, at their shared focus, a source of low-frequency ultrasound at the difference frequency of the carrier waves. Here, modulated focused ultrasound (mFU) allows application of low-frequency ultrasound to small regions of the brain. To test the efficacy of mFU and the relative contributions of its constituent parts, we visually recorded evoked movements while transcranially stimulating different parts of mouse brain w under light anesthesia. The mFU protocol followed, where possible, existing published ultrasound protocols, using a difference frequency of 500 kHz. mFU induced each of unilateral and bilateral motor function that varied by location, intensity, and the inclusion or exclusion of the low-frequency temporal modulation of the high-frequency carrier wave.

## Methods

### Ethics Statement

All animal procedures were approved by the University of Washington Institutional Animal Care and Use Committee (IACUC).

### Animal Model and Anesthesia

Male C57BL/6 mice, age 8–12 weeks, weight 22–27 g, were anesthetized with a mixture of ketamine and xylazine [11]. Ketamine and xylazine concentrations administered were 87.5 mg/Kg and 8.75 mg/Kg, respectively, with supplemental doses administered as needed to maintain anesthesia during longer experiments. A heating pad set to 100’F maintained the body temperature of the mice while they were under anesthesia. Hair was removed from the top of each mouse’s head via shears and application of Nair® (Church and Dwight Co., Inc., Princeton, NJ, U.S.). Toe and/or tail pinches were given every 10 min to assure mice stayed reactive to such stimuli but were otherwise quiescent. Aquasonic (Parker Laboratories, Inc., Fairfield, New Jersey, U.S.) ultrasound coupling gel was placed on the skin to ensure proper transmission. Brain tissue for histological analysis was collected after perfusion with 1 mL of paraformaldehyde within 5 min of the last experimental trial. Mice not used for histological analysis were euthanized with pentobarbital, concentration 400–500 mg/Kg.

### Ultrasound Sources

**Figure 1:**
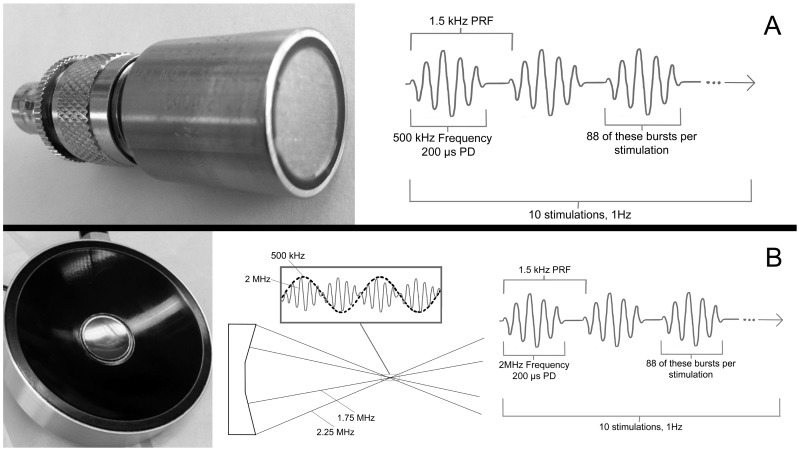
Transducers and their associated ultrasound emissions. (A) Ultran planar ultrasound transducer with corresponding waveform representation. (B) Sonic Concepts focused ultrasound transducer (black annulus with filled hole) with corresponding waveform representation.

Two transducers were used for experiment trials: a single-frequency planar, ultrasound source and an effectively multi-frequency, focused ultrasound source. A pulsed, single-frequency UNMOD protocol using a planar piston transducer (Ultran Group, Ultran GS500-D13, State College, PA, U.S.; Fig. 1A) was developed following King et al. [5] and Tufail et al. [11]: 88 bursts of 500-kHz ultrasound, each of length 200 µs, at a pulse repetition frequency of 1.5 kHz in a 1-s interval.

A focused UNMOD protocol was designed to overlap the UNMOD protocol using the planar Ultran transducer. A dual element, coaxial, confocal and circular transducer and associated matching networks (H-148, Sonic Concepts, Woodinville, WA, U.S.) with a filled, central opening were used (Fig. 1B). Two Agilent Series 33220A 20-MHz function generators (Agilent Technologies, Santa Clara, CA, U.S.), controlled by a third Agilent function generator, drove two ENI brand model A150 55-dB amplifiers (Electronic Navigation Industries, Rochester, NY, U.S.) that, in turn, powered each of the two transducers within the focused transducer. A LeCroy Oscilloscope (Waverunner LT344,Teledyne LeCroy, Chestnut Ridge, NY, U.S.) monitored the voltage entering each transducer element. During the focused ultrasound for UNMOD studies each element of the transducer was driven at 2 MHz. To study the effects of mFU – the vibro-acoustography technique – one element of the focused transducer was driven at 1.75 MHz and the other at 2.25 MHz, producing a difference frequency of 500 kHz at the focus. Otherwise, the mFU parameters mimicked those of the planar transducer at 500 kHz.

For both ultrasound methods, one trial consisted of ten applications of this ultrasound protocol and was completed in approximately 10 s.

**Figure 2:**
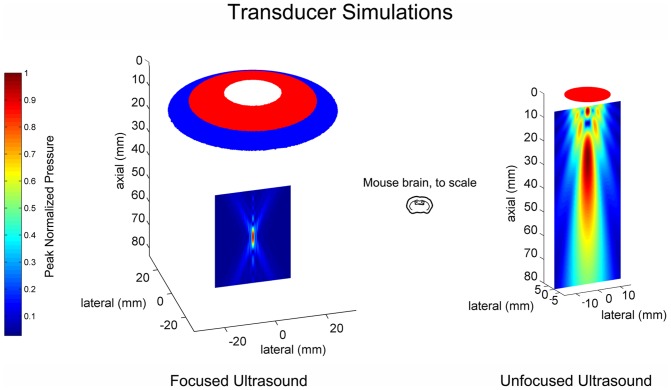
Ultrasound pressure fields for our devices. Simulations of focused and planar ultrasound beam plots, with mouse brain for comparison.

The length and width of the mFU transducer focus, measured at the ‘half pressure’ value during simulations in water, is 8 mm in the axial direction and 1.5 mm in the lateral direction, yielding approximately 0.015 mL. For the planar ultrasound device, the broad ‘focus’ measured greater than 40 mm in the axial direction and 12 mm in the lateral direction, yielding approximately 4.5 mL (Fig. 2).

### Ultrasound Calibration

The experimental transducers were placed in a tank filled with degassed and deionized water and the active tip of a calibrated needle hydrophone (HNR-1000, Onda Corporation, Sunnyvale, CA, U.S.) was then placed at the focus of each of the two elements of the dual-element transducer and at the point of maximum pressure from the planar transducer. To verify that the voltage into each element of the dual-element transducer produced the same pressure at a given frequency, thereby insuring that when both elements were run simultaneously each contributed equally to the pressure, peak positive pressure was measured with each element running individually. Each of the two elements produced half the peak pressure that was measured when both elements were combined; thus, we fine-tuned the voltage required by each element to produce half the peak pressure of a predetermined value.

To calibrate the Ultran transducer the tip of the needle hydrophone was placed at the center of its planar face and moved axially to locate its broad, maximum peak pressure at roughly 2 cm from the face. For both experimental transducers the spatial peak temporal average intensity (I_spta_) is reported.

### Ultrasound Deployment

The concave side of the FUS transducer had on its distal surface a hollow, plastic cone with a large opening covered with 0.1524-mm thick latex to allow transmission of ultrasound. Between the transducer face and the latex covering, the transducer housing contained degassed and deionized water. The transducer housing was attached to a metal arm connected to a micro positioner. The positioner stage acted as a 3D-coordinate grid – allowing transducer movement through the necessary x–y, x–z, and y–z planes with sub-millimeter precision. Green laser lights attached to the transducer housing facilitated precise positioning of the transducer focus. A red light emitting diode (LED) attached to a small ruler placed near the front of the animal and within view of a video camera indicated the time of ultrasound application. Body movements and the blinking red LED were recorded with a Nikon D3200 camera (Nikon Corporation, Tokyo, Japan). A plastic, 3D-printed support positioned the mouse to allow the front paws to hang, keep the head secure, and prevent the body from rolling side-to-side. The green lasers were aligned on the surface of the skin, placing the geometric focus of the ultrasound in the same location. The height was marked on the micro-positioner, the transducer moved away from the skull, and ultrasound gel applied. Ultrasound was focused to a position 5 mm below the skin surface – the target depth chosen after imaging the mouse head with a diagnostic ultrasound device.

The planar transducer was clamped directly to the micro positioner and the transducer was applied directly onto the mouse scalp coupled with ultrasound gel.

#### Ultrasound Administration – Planar (Ultran) Transducer

**Figure 3:**
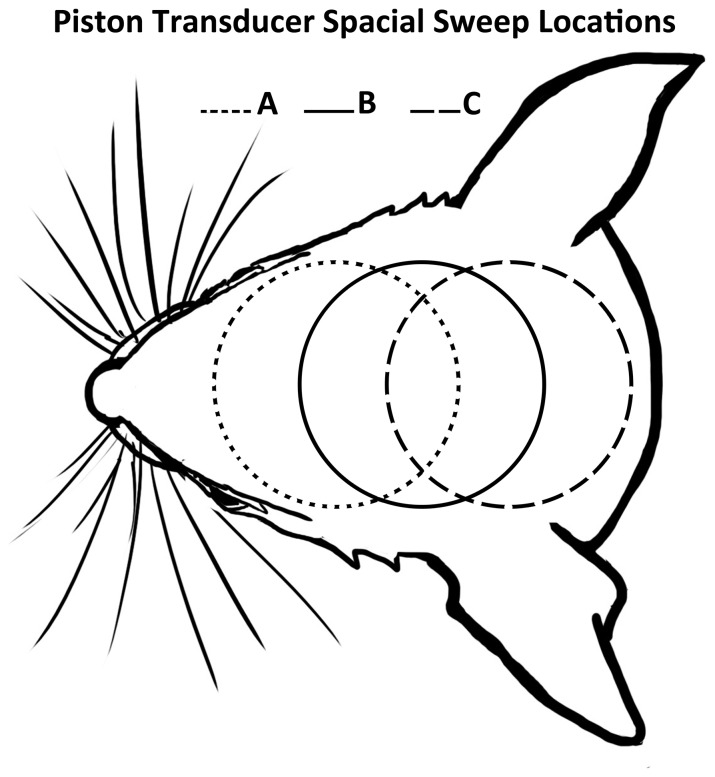
Superficial projection of intra-cranial stimulation regions crated by planar ultrasound. Three regions of quantitatively different stimulation responses created by a sweep of our planar ultrasound device.

Planar ultrasound was applied in a rostral to caudal sweep along each mouse skull midline with 3 mm between stable positions A, B, and C (Fig. 3). One ultrasound trial was administered to each of the three positions with an interval of about 5 min between trials. I_SPTA_ was 5.25 W/cm^2^. During pilot studies ultrasound was applied continuously and was swept slowly from one position to another. No significant motor activity differences were observed for small movements of the transducer (1 mm scale), but rather only when traversing large portions of the mouse skull (front, middle, rear, on scales of several millimeters). Another series of trials at position B (the mid-sagittal region) were conducted with I_SPTA_ varied from 0.15 to 5.25 W/cm^2^ by changing only the peak pressure and maintaining the temporal pattern.

#### Ultrasound Administration – mFU at Various Intensities

For a separate group of mice an anatomical position was determined for each mouse where a 10-s application of mFU via the standard protocol (Fig. 1A) caused robust motor movement. Without moving the source mFU was reapplied for sets of ten more stimulations, varying the number of bursts or pulse duration to decrease the intensity, and recording the evoked movements for each combination.

#### Ultrasound Administration – mFU and FUS Applied to Separate Mice

**Figure 4:**
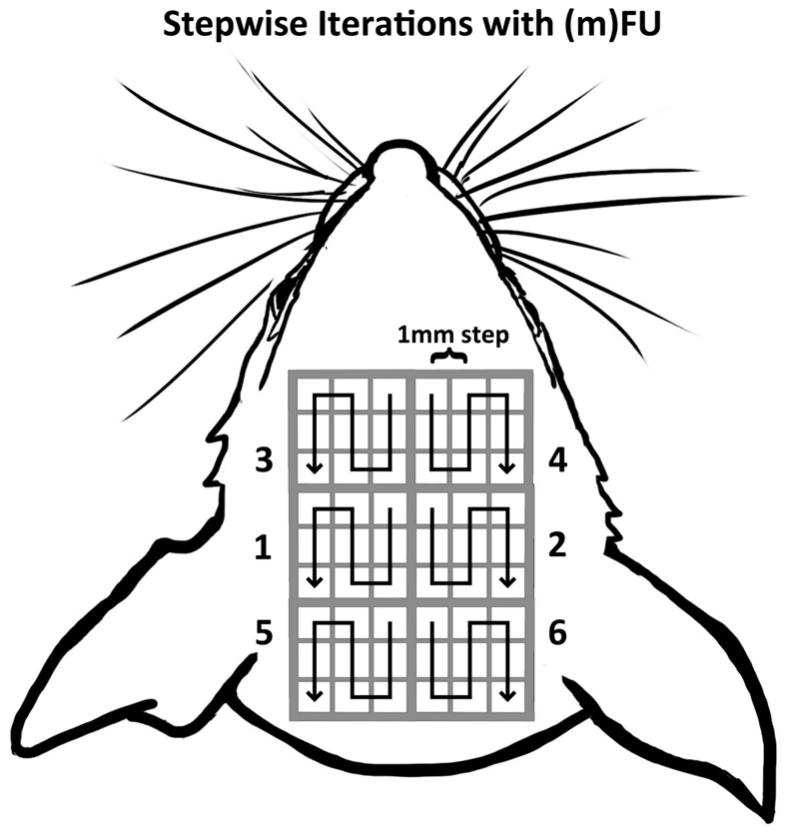
Superficial projection of intra-cranial stimulation regions created by mFU and FUS. The 54 squares represent the superficial projection of individual, intra-cranial stimulation regions, with centers separated in 1 mm increments. Trials began in region 1 and concluded in region 6, following the arrow within each region.

The focused transducer was moved incrementally in steps of 1 mm along the top of the mouse skull through six regions each measuring 3×3 mm spanning the bregma to the lambda sutures in a manner that emphasized the parietal region. Each of these six regions was divided into a 3×3 grid to create a 54-element stimulation grid with 1-mm resolution. Each portion of this 54-region grid was stimulated with ultrasound in the same order for each mouse (Fig. 4). FUS or mFU application was delayed by five minutes between each of the six major regions. Within a region, the trials were paused only to move the transducer to each new location between applications.

#### Ultrasound Administration – mFU and FUS Applied to the Same Mice

The standard protocol was amended to five stimulations per position instead of ten. The locations of the stimulations remained the same, with the exclusion of the two most rostral grids (Fig. 4, eliminating grids 3 and 4). Within each grid the same paths were followed, except for beginning in the lower right large square (grid 6) and circling clockwise through the three remaining squares (hence to grid 5, then grid 1, then finishing at grid 4) after the stimulations were complete. We performed this study in this fashion motivated by our first results, with mFU alone or FUS alone, where as we report below stimulation of regions five and six – the most caudal regions – produced the majority of the observed induced motions. In each position mFU was applied and any motor movements noted, then the application was switched to FUS by equalizing the carrier frequencies. The trial was repeated if any motor movement with either mFU or FUS was observed. If no movement was observed, the trial advanced to the next location.

### Data Acquisition and Analysis

All experimental trials were captured on video using a Nikon D3200 camera complemented by hand-written notes collected by a minimum of two lab members for each trial. This allowed incorporation into the subsequent analysis of the videos observations taken from three different perspectives. Three people reviewed the videos of each experimental trial multiple times while referring to the hand-written notes.

#### Definition of a motor movement robustness scale

**Figure 5:**
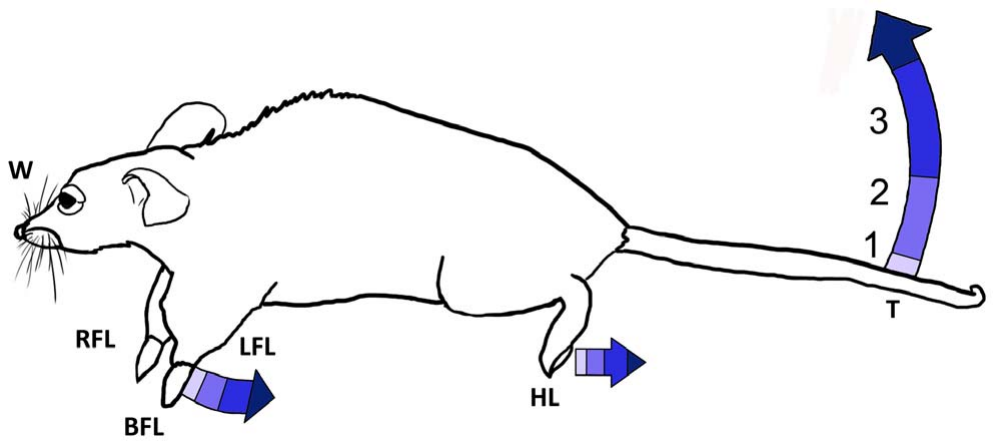
Ordinal robustness scale for motor movements. A value of one represents minimally observable motion while a value of three represents the largest motions regularly observed.

Common movements observed over multiple trials were left and right paw raises (individually or together), paw extension, tail flicks or extension, and whisker flicks. A quantitative measure of the extent of these motions – their ‘robustness’ – was created with an ordinal scale of 0–3 (Fig. 5).

A value of zero was assigned for no observed movement correlated with application of ultrasound, only movement associated with the breathing of the mice.

A value of one was assigned for observed faint movements, at least a twitch, with amplitude of up to 1 mm. Paws would twitch up or down, hind legs would briefly flex, and the tail would flick, usually upwards. At this degree, generally only the tip of the tail would move.

A value of two was assigned for observed moderate movements with amplitudes as high as 5 mm. Also, partial tail extension would occur, usually lasting a little over one pulse duration.

A value of three was assigned for observed strong movements of 1 cm or greater in amplitude and represented the largest motions regularly observed.

Less than one percent of the time larger, strained, or rare movements were observed, including limb extension and multidirectional tail movement including spinning. These may have been a sign that the light anesthesia required reapplication; in these cases additional anesthesia was administered to complete the experimental protocol.

#### Qualitative measures of induced motor activity

A qualitative comparison of mFU with FUS focused on those positions in a given mouse where mFU and FUS could induce motion each time they were applied. In addition to robustness, defined previously, ‘fluidity’ was defined as a measure of the sharpness or crispness of the observed movements as observed grossly, and ‘repetition’ as a measure of the consistency of each action within a trial. A binary determination was made whether mFU or FUS elicited the stronger response at the same anatomical location in the same mouse according to how appropriately they fit the categories. If mFU and FUS could not be differentiated the site was labeled as no discernable difference.

## Results

### Planar Ultrasound Device Intensity Sweep

**Figure 6:**
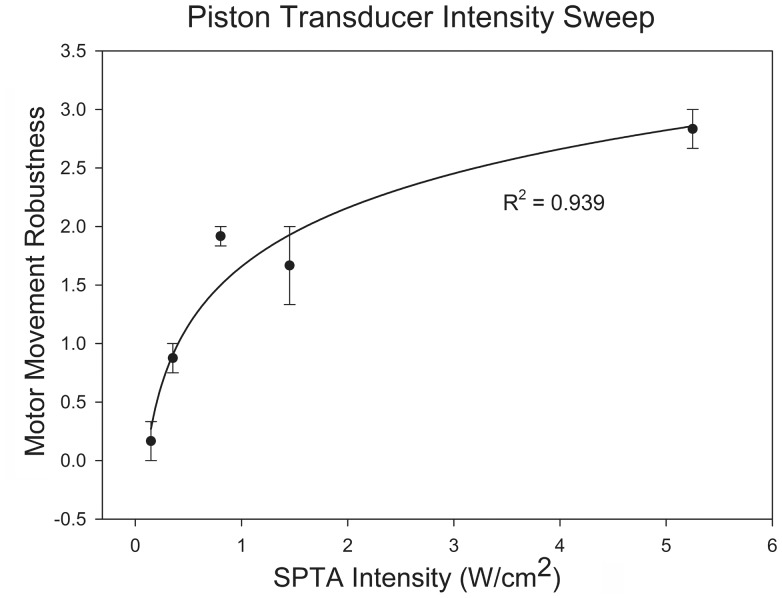
Intensity of mFU stimulation versus robustness of associated observed motor movement. Curve represents a logarithmic fit. (N = 3).

Figure 6 shows a logarithmic fit (R^2^ = 0.939) between the ultrasound intensity and the average degree of motor movement caused by the stimulation at that intensity. Results from three mice demonstrate that the greater the ultrasound intensity the larger the induced movement by the ultrasound. Tail movements and bilateral (only) movement of legs and whiskers were observed.

### Planar Ultrasound Device Spatial Sweep

**Figure 7:**
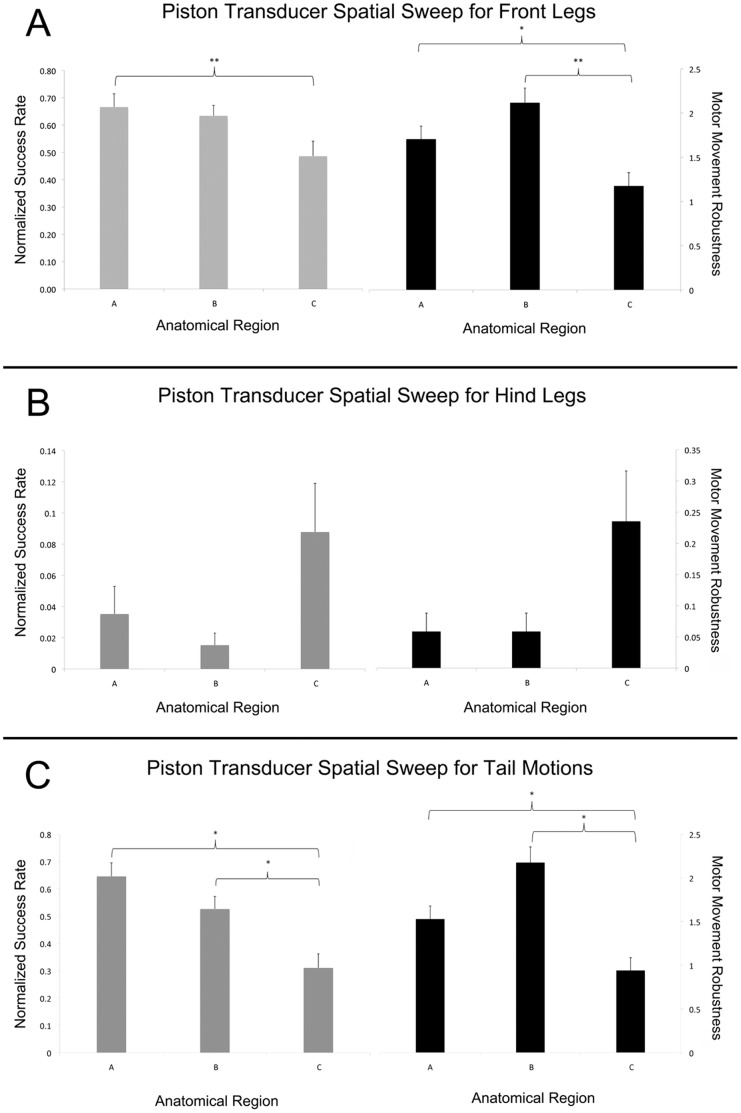
Success rate and robustness of movements induced by ultrasound from a planar source. We report these values for (A) for front legs (B) for hind legs and (C) for tail. The success rate was normalized to a value of 1 at 100% success (10/10 motions). Note the different vertical scales for each graph. One-way ANOVA test was run, * refers to significant difference (p-value <0.05), ** refers to approaching significance (p-value <0.1). (N = 6).

Tail movements and bilateral (only) movement of legs and whiskers were observed using the planar ultrasound device with six mice. There was an overall decrease in the number of front leg and tail movements as the planar ultrasound transducer moved from region A (caudal) to region C (rostral) of the mouse brain (Fig. 7A–C). Region A had the highest average success rate for front leg and tail movement, while region B had the highest average robustness. A significant difference in the robustness and success rate of front paw activity between regions A and C was also observed (Fig. 7A), as well as a difference in robustness between regions B and C. Analysis of hind leg movement showed no significant difference in success rate or level of robustness for the three regions (Fig. 7B). There was, however, a significant difference in success rate and robustness of movement for tail stimulations between regions A and C as well as B and C (Fig. 7C).

### mFU Applied with Variable Intensity

**Figure 8:**
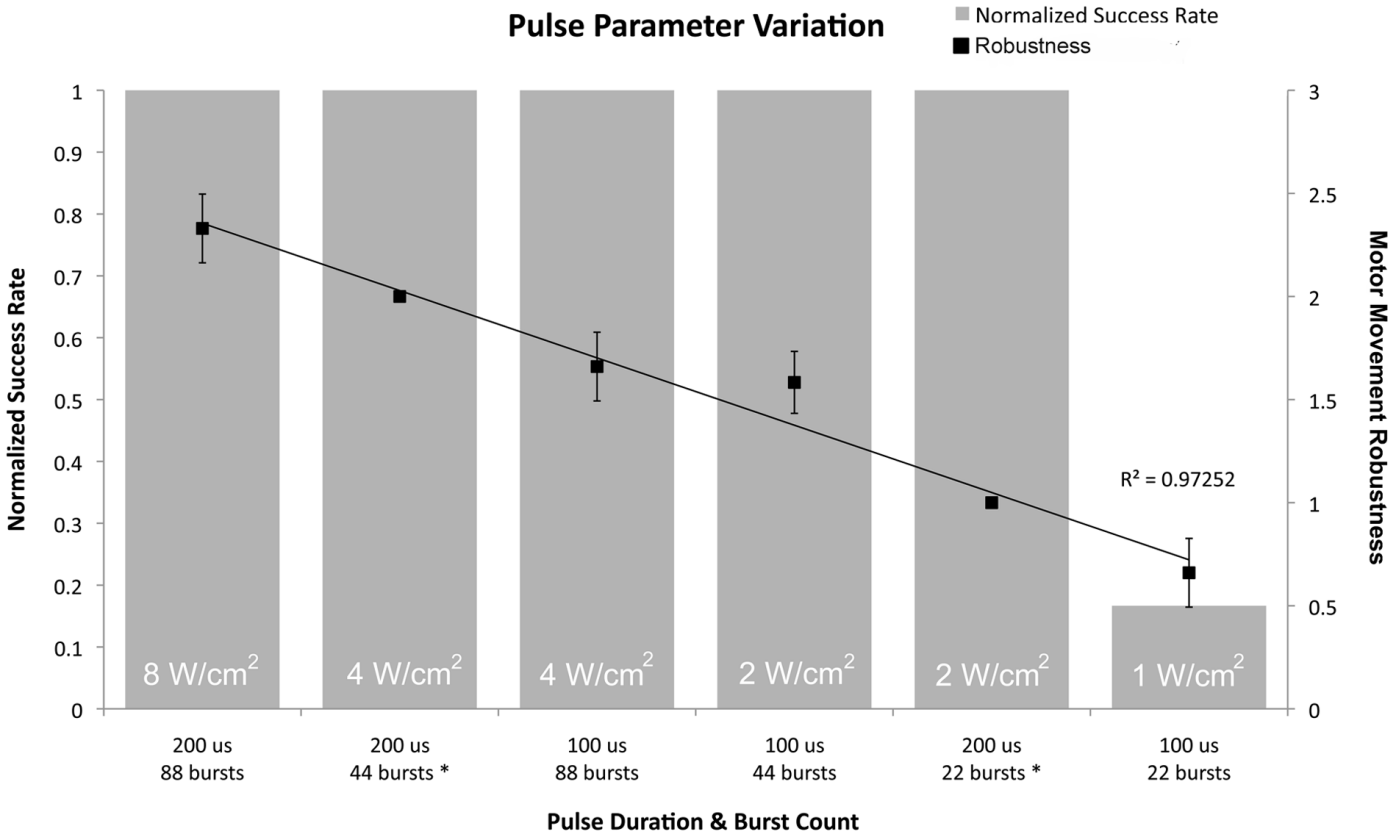
Intensity sweep of modulated focused ultrasound. Intensities shown are spatial peak temporal average values. Robustness data were linearly fit with R^2^ = 0.97252.

In trials with three mice, the intensity of the standard ultrasound protocol was decreased by changing the number or duration of pulses. Motor responses were induced until reaching 1 W/cm^2^ and robustness of the induced movements decreased linearly with intensity (Fig. 8).

### mFU and FUS Applied to Separate Cohorts of Mice

**Figure 9:**
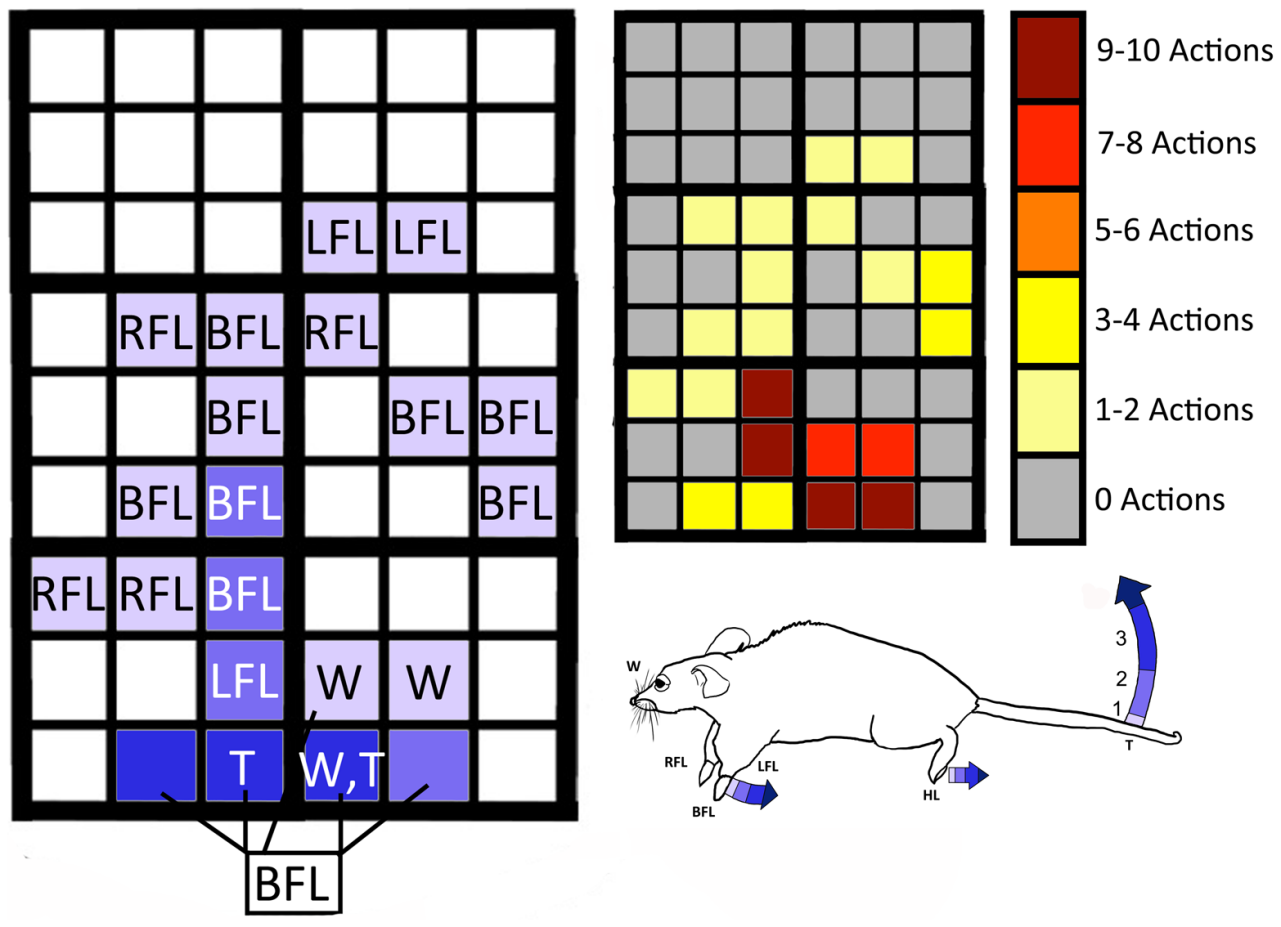
Example of motor robustness and success rate values generated by mFU applied to one mouse. (Left) Motor robustness and type of movements observed for one mouse with application of mFU. (Right) Corresponding success rate, out of a possible ten actions. (BFL) both front legs, (RFL) right front leg, (LFL) left front leg, (T) tail flick, (W) whiskers.

**Figure 10:**
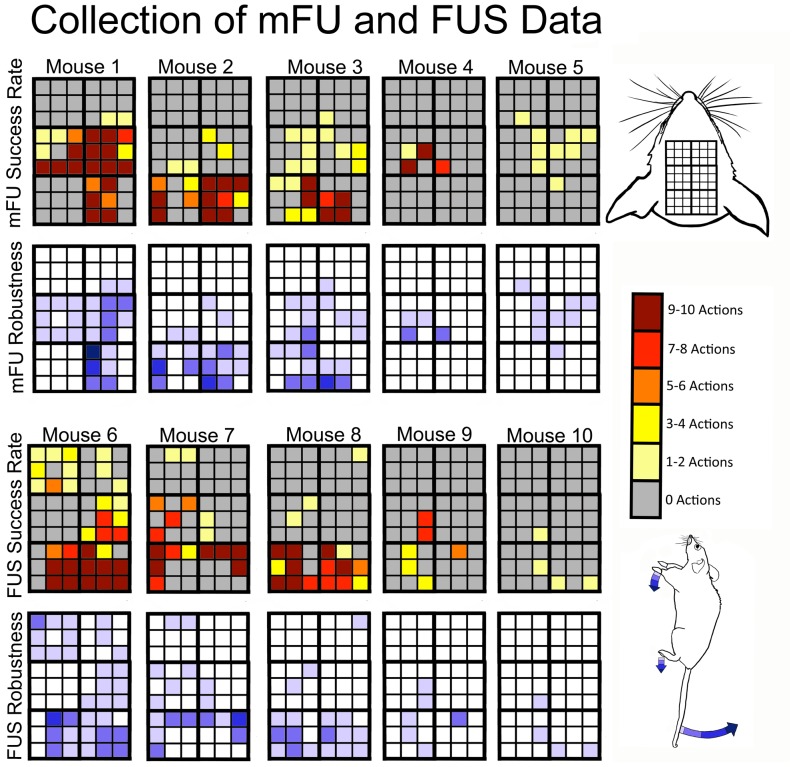
Motor stimulation data for all ten mFU and ten FUS mice. The red-shaded colors denote number of actions with a maximum value of ten while the range of blue shades quantifies the size of motion. The top two rows show results due to mFU alone while the bottom two rows show results due to FU alone. Results are displayed with the highest success rate to the left and lowest success rate to the right.

Evoked motor responses were sensitive to position of ultrasound delivery with a spatial resolution of ∼1 mm. Moreover, the fraction of successful stimulation events (of ten repetitions) varied considerably (Fig. 9). Some of the five mice for each of the FUS and mFU trials showed minimal induced activity while others showed substantial induced activity under each mFU and FUS protocol (Fig. 10). When averaged across all mice and all positions, however, there was comparable success between the ability of mFU and FUS to induce movement: mFU induced some type of motor activity in 75 out of 270 stimulations (27.78%) and FUS induced observable motor activity in 77 out of 270 stimulations (28.52%; Table 1).

**Table 1:** BFL - Both front legs, LFL - Left Front Leg, RFL - Right Front Leg, (Left) Right Front Leg, T-Tail, HL - Hind Legs, W-Whiskers.

	BFL	LFL	RFL	T	HL	W
mFU	15.56%	2.59%	4.81%	8.52%	1.11%	4.07%
FUS	18.15%	3.33%	3.70%	17.04%	0.00%.	0.37%

**Figure 11:**
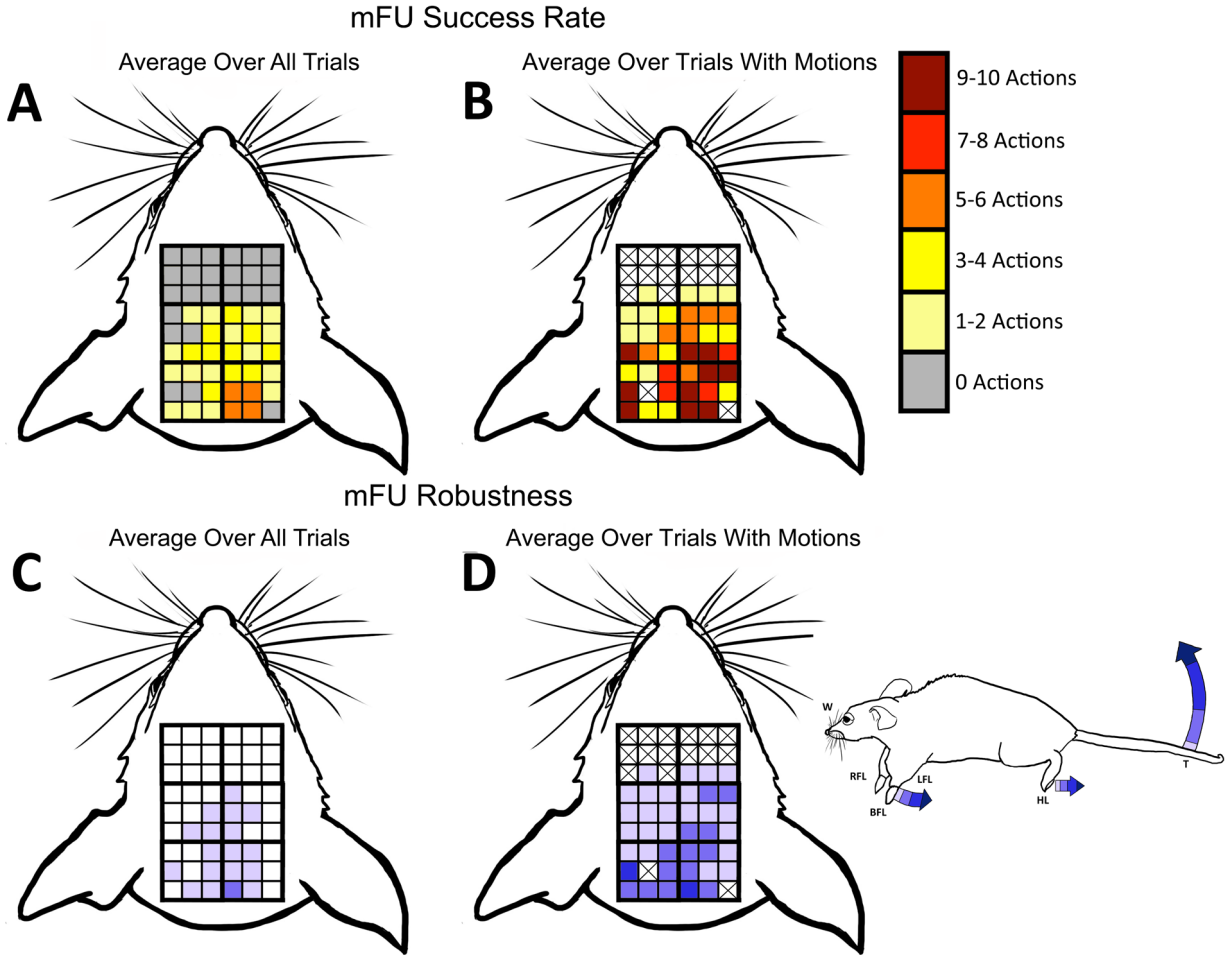
Metrics for successful stimulation by mFU. Measures of robustness and success rate of induced motions by mFU averaged over 5 mice. The X’s in (B) and (D) indicate that no movement in those regions was ever observed. X’s are not shown in (A) or (C) because there is the possibility that the data rounded down to 0 (<0.5 actions).

**Figure 12:**
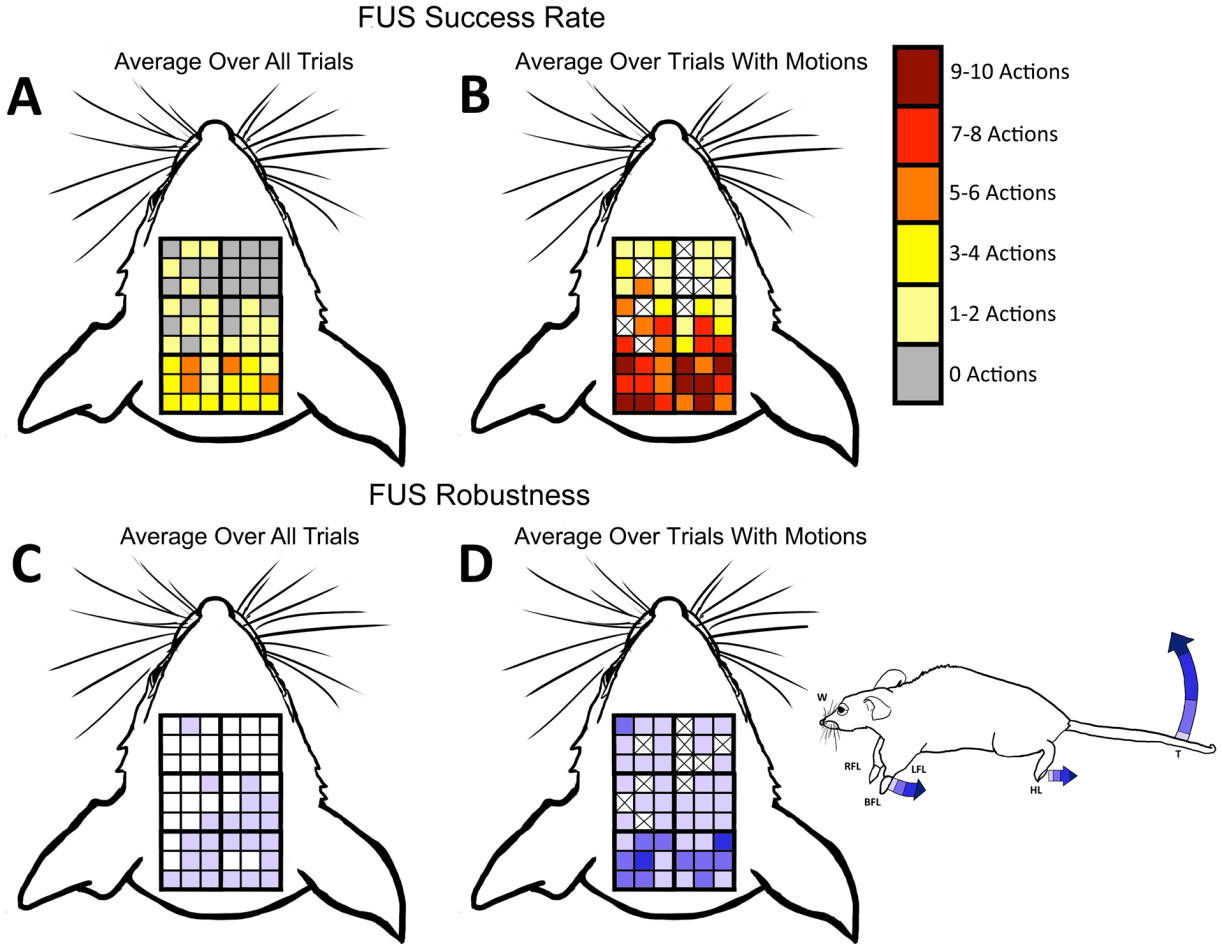
Metrics for successful stimulation by FUS. Measures of robustness and success rate of induced motions by FU averaged over 5 mice. The X’s in (B) and (D) indicate that no movement in those regions was ever observed. X’s are not shown in (A) or (C) because there is the possibility that the data rounded down to 0 (<0.5 actions).

Both protocols also caused the same range of motor movement. Averaging over the behavioral results at each grid point over all five mice in the experiments yields low success rates (Fig. 11a,c; Fig. 12a,c). This analysis, however, obscures the fact that when ultrasound induced movement, it did so for a large percentage of the stimulations. To account for the large variance of results for movement induction, data are also reported only for trials in a given position that showed successful stimulation (Fig. 11b,d and 12b,d). The highest success rates for each of mFU and FUS were observed in positions 5 and 6. This is the most posterior location over the parietal region of the brain, and also where the most robust induced motions were observed. In contrast, the most anterior area – regions 3 and 4– showed the lowest percentage of induced motions as well as the least robust movements.

### mFU and FUS Applied to the Same Mice

**Figure 13:**
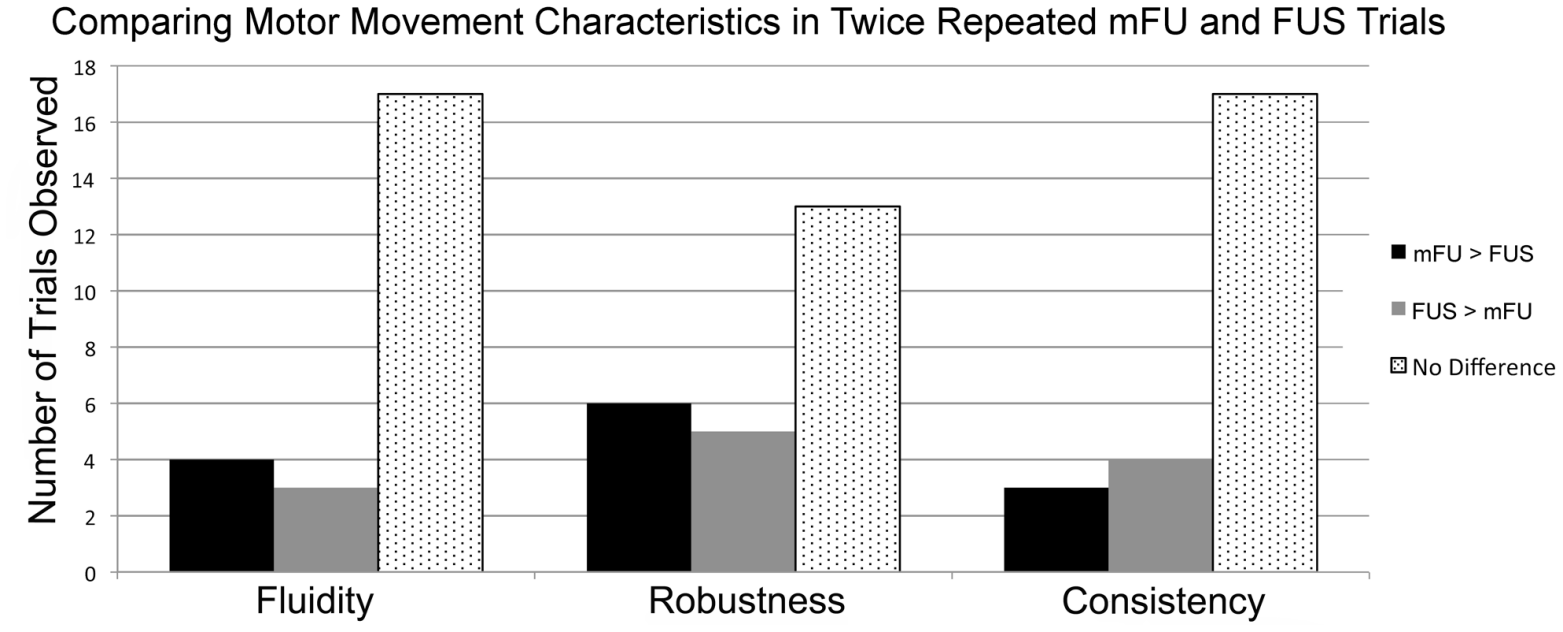
Qualities of movement induced by mFU versus FU applied successfully to the same region of the same mouse. Comparison between different measures of motion induced by each of mFU and FU applied to the same mice for cases where each protocol elicited a motor response twice in succession. Relative fluidity, robustness, and repetition of the movements are evaluated for 24 successful stimulations across three mice.

Trials with mFU and FUS applied to the same three mice resulted in 458 total stimulations, with 99 eliciting an observable motor response. Out of a possible 180 positions for stimulation, induced movement was observed in 37 (20.56%) of those locations with mFU, FUS, or both modalities. Of these 37 locations, 13 showed movement induced by both methods. Only mFU or only FUS stimulation induced movement in the other 24 locations, though these results varied significantly between individual mice. The distribution totals of these combinations are given in Table 2. Qualitative analysis of those 13 positions where mFU and FUS induced motion each time they were applied detected little difference between mFU and FUS in terms of robustness, fluidity, and repetition (Fig. 13).

**Table 2:** Distribution of successful stimulations in combined mFU and FUS mice.

Total locations where movement was observed	37
mFU &/or FUS, at most one seen twice	24
mFU & FUS, each seen twice	13
Total locations stimulated	458

### Histological Analysis

Hemotoxilin and eosin, and cresyl violet stained sections of brain were analyzed for damage associated with ultrasound protocols. All histology samples show unaffected brain with no interesting artifacts (data not shown).

## Discussion

Studies were performed with both poorly focused and very focused (plus multi-frequency) transducers to compare their ability to induce movement in response to transcutaneous/transcranial ultrasound applied to the brain. Application of ultrasound from a planar source to mouse brain induced motor movements whose amplitude scaled with peak pressure (Fig. 6). This differs from previous results reported by King et al. [5], where all or nothing responses were reported. This may be due to our use of ketamine/xylazine versus isoflurane. Inhalation of isoflurane inhibits the transmission of motor evoked potentials through the brainstem [12]. Thus, centrally targeted motor stimulation may require relatively intense ultrasound stimulation to produce an observable peripheral effect. In contrast, ketamine/xylazine has been shown to have only limited effects on peripheral sensory or motor conduction [13]. In pilot studies using isoflurane (unreported here) we observed all or nothing responses similar to those reported by King et al. [5].

Ultrasound from the planar source produced a low-frequency (500 kHz) rapidly pulsed sequence (88 pulses at a PRF of 1.5 kHz, each pulse lasting for 200 µs) with an intensity of 5.24 W/cm^2^, following the temporal structure of the ultrasound protocol demonstrated by Tyler et al. [6] and intensity of King et al. [5]. This stimulation source produced largely uniform, repeatable, and exclusively bilateral motor responses (primarily tail and front leg motion; minimal hind-leg motion) over a range of transducer locations spanning 4–6 mm in a rostral to caudal direction (Fig. 3 and 7). Manipulation of the location of ultrasound from a planar source served as a first step towards showing anatomical specificity of UNMOD. Although the sweep was limited, it produced differential robustness of induced activity, though not type of induced activity, in a manner that corresponded with three different anatomical regions located several millimeters apart.

The same low-frequency ultrasound protocol was used with the modulated (at 500 kHz) high-frequency (2 MHz) focused ultrasound made possible by vibro-acoustography. This mFU protocol generated different motor responses in mice when changing the position of ultrasound application by as little as 1 mm (Fig. 9). Both the type of evoked movement, as well as the robustness often varied with mFU probe location. Unilateral paw movements were observed at 28% of locations tested, with the remainder of the evoked paw movements consisting of bilateral motions. In contrast, but in agreement with other studies [3]–[5], we observed only bilateral paw motions generated by the planar transducer. Nonetheless, these results were highly variable both within a given mouse and between mice (Fig. 10).

Kim et al. [8] report in their studies of neuromodulation that the ultrasound focus was diameter 3.5 mm and length 6.2 mm at full width half maximum pressure (with an associated volume of approximately 0.06 mL). By comparison, the vibro-acoustography studies described here had diameter 1.2 mm and length 8 mm (with an associated volume of approximately 0.015 mL). The ultrasound carrier frequency of 2 MHz was also higher than that used previously [7], [8], but is still sufficient to transmit trans-temporally through a human or primate skull. If needed, however we can in principle modify our UNMOD protocol via mFU to allow for transmission across thicker regions of the skull by making use of a lower carrier frequency of ∼1 MHz, though we have not tested this in practice. Also, King et al. [5] and others have shown that low frequency UNMOD is effective at frequencies as low as 250 kHz. We can readily apply such low frequency UNMOD protocols within, however, a much smaller volume of brain accessible to single-frequency devices, using the vibro-acoustography paradigm. Indeed, exploration of the potential efficacy of UNMOD at frequencies in physiologically relevant bands (tens to hundreds of Hertz) is possible via our methodology. Supporting this, Greenleaf and colleagues [9], [10] have deployed vibro-acoustography paradigms for imaging purposes with difference frequencies as low as 7 kHz with no intrinsic reason why they could not go lower. Exploring UNMOD at very low difference frequencies represents an important target of our next research efforts.

What constituents of the mFU technique are most strongly correlated to the observed biological effects? Holding the spatial and temporal peak pressure, and the pulse repetition frequency constant while varying the pulse length and number of pulses per stimulation acts to decrease the spatial peak temporal average intensity. Over a significant range of spatial peak temporal average intensity, mFU produced comparable motor responses, though the magnitude of those responses declined linearly as intensity decreased. This linearity of movement response contrasts with the non-linear responses to varying levels of electrical stimulation delivered to the brain [14], likely due to underlying nonlinearities in current spread and resulting spatial summation of electrical stimulation [15]. Linear activation of neural tissue may be a key advantage of ultrasound stimulation, as non-linear activation of the peripheral nervous system, for example, has limited the clinical utility of functional electrical stimulation [16], [17].

The vibro-acoustography technique reported here also introduces higher frequencies into the ultrasound stimulation protocol than have been considered in previous UNMOD studies. The anatomical specificity, robustness, fluidity, and repetition of motor activity induced by mFU versus FUS were similar (Fig. 10–13). Large variances in observations for mFU stimulation were matched by those for FUS stimulation alone. This observation highlights the likely role of the radiation force found in each pulse of ultrasound (one of the constants between mFU and FUS) as a significant contributor to the observed effect, with the pulse repetition frequency of its application now meriting additional scrutiny.

There were clear differences, however, in the ability of mFU versus FUS to produce a motor response when applied to the same portion of brain of the same mouse. This suggests that the pulse-associated radiation force does not represent the sole means of producing UNMOD with ultrasound. While at times mFU and FUS worked comparably well at a given location, they often did not (Fig. 10–12; Table 2). This difference suggests that the low-frequency component to mFU does contribute in a unique way to UNMOD, and is consistent with our direct observations that FUS alone was not always sufficient to induce motor responses. A greater understanding of this difference will require additional work, including examination of the specific anatomical targets that are receptive to mFU versus FUS. For example, while electrical stimulation of the central nervous system is known to activate axons at lower stimulus intensities then neuron cell bodies or their dendrites [18], the mechanism by which ultrasound activates neural tissue is currently unknown, and may depend upon the presence or absence of a low frequency component of ultrasound.

Finally, both the largely bilateral movements evoked using mFU, and the large variance in induced motion both within and between mice, suggest that multiple deep brain structures are activated, with little direct stimulation of unilateral motor cortex. The regions of the brain likely stimulated during these UNMOD studies with mFU include, but are not limited to, the cerebral cortex, basal forebrain, midbrain (e.g., red nucleus and substantia nigra), hypothalamus, thalamus, hippocampus, cerebral cortex, basal forebrain, caudate striatum, and corpus callosum. All of these structures are involved in motor movement either directly or indirectly. If ultrasound stimulation preferentially activates axons at lower intensities than cell bodies (as is the case for electrical stimulation), the predominance of bilateral movements may originate from activation of large axon tracts such as the corpus callosum, which functions in part to coordinate motor activity between the two hemispheres. In addition, the red nucleus integrates information from the contralateral cerebellum and ipsilateral motor cortex, so its activation (either directly or indirectly) may result in bi-lateral movements of the upper forelimbs, although perhaps most naturally in an alternating pattern such as observed during gait, which we did not observe. The relay circuits of the thalamus may also contribute to the evoked activity, although cortical motor projections are largely lateralized with the exception of a minority of pre-frontal projections [19]. Most probably, the sphere of activation of even focused ultrasound directly activates bi-lateral structures in the mouse brain, suggesting it is necessary to perform larger animal studies to determine the stimulation effects on individual brain areas.

### Limitations

The greatest limitation to this project is the size of the mouse brain relative to the focal zone of the ultrasound sources. Even for FUS and mFU, the roughly 8-mm focal length and 1.5-mm focal width of the ultrasound’s highest intensity region is large enough to simultaneously stimulate several anatomically distinct portions of the brain. This limitation may be overcome with a larger animal model in tandem with intra-operative brain mapping. In addition, it is a worthwhile engineering task to produce ultrasound devices with a smaller focus.

Several protocol parameters were not explored. These include the pulse repetition frequency of 1.5 kHz, the modulating frequency of 500 kHz, as well as a wider range of pulse lengths and number. Of particular interest would be pulse repetition and modulating frequencies that fall within the frequency band associated with natural physiological brain processes. This would also bring the project closer to direct comparison with neuromodulation by electro-stimulation. Typical frequencies measured by EEG are 8–12 Hz, “alpha” waves; 18–26 Hz, “beta” waves; and >30 Hz, “gamma” waves [20]. Vibro-acoustography techniques could be used to explore the potential effects of spatially compact but very low-frequency ultrasound signals on brain function.

Two of the many phenomena associated with these low-frequency signals within brain are event related desynchronization (ERD) and event related synchronization (ERS). ERD refers to the somatotopically defined decreases in the low-frequency band that occur during motor movement, or decreases in the correspondence between parts of the body and specific regions of brain, while the opposite is true for ERS. Desynchronization can be observed in the idling beta activity peaking around 20 Hz, for example, when an individual processes sensorimotor information or performs a motor task [21]. During these same activities, ERS can be observed by an increase in spectral power in the gamma frequency range [20]. Perhaps mFU with a modulating frequency below 30 Hz could modify the normal ERD or ERS processes by increasing or suppressing the phenomena.

Acute histological analysis after UNMOD trials showed damage-free brain. Repeated application of mFU and FUS yielded reproducible results, suggesting that brain function was not altered focally or acutely. In addition, the protocols used spatial peak temporal average intensities within the range that others have reported to be both efficacious and safe [5]–[7].

Yoo et al. [7] employed fMRI to observe alteration of brain function in deeply anesthetized rabbits via UNMOD at much lower intensities than we used. By design, they did not observe any grossly observable motor function. Because of the observations of Yoo et al., and our observations of induced motion at I_SPTA_ values of 1 W/cm^2^, near the FDA limit of 0.72 W/cm^2^, we are optimistic that the mFU embodiment of UNMOD may be deployed within FDA limits for ultrasound. These FDA limits on ultrasound intensity, thermal index, mechanical index, etc. are defined for frequencies greater than or equal to 1 MHz, however. Therefore continued attention to safety is warranted, along with use of a range of observable correlates to successful UNMOD. Examples include fMRI, or in future animal studies the use of fine-wire electromyograms (EMG) to measure potentials across different muscle groups in the legs, tail, and other anatomical structures. This would allow measurements of smaller motor excitations than are visible as gross movements.

## Conclusions

Transcranial ultrasound applied to the brain can transiently and nondestructively activate it using a range of parameters and devices. Previous research used pulses of low-frequency (250–700 kHz) ultrasound with spatial peak temporal average intensities (I_SPTA_) of 0.1–10 W/cm^2^, emitted from transducers that insonified large volumes of mouse brain relative to our system, and all with a single carrier frequency of ultrasound. Typical observations to date include induced motor activity timed to the delivery of ultrasound, but without the ability to vary the type of activity. This study seeks to add anatomical specificity to current neuromodulation practice through the use of focused ultrasound (FUS) by itself, or a modulated variant (mFU). ‘Modulated’ refers to adding complex low-frequency temporal modulation (500 Hz here) of the higher frequency (2 MHz), pulsed and focused waveform in the manner of vibro-acoustography. With lightly anesthetized mice as test subjects, regions of brain were stimulated with 1-mm resolution. Each of mFU and FUS alone were sufficient to induce motor activity, though not always at the same anatomical location. Induction of a variety of motor functions varied by intensity (0.1–5.0 I_SPTA_), and by the inclusion or exclusion of the low-frequency temporal modulation of the high-frequency carrier wave. Responses were spatially selective, with diverse movements (both unilateral and bilateral) evoked by both ultrasound methods often at adjacent stimulation locations separated by only 1 mm. In future work we will seek to determine the relative efficacy of mFU versus FUS, to further refine the portions of the UNMOD paradigm most closely tied to its efficacy, and to study focal stimulation of central nervous system structures at the very low frequencies that arise naturally within brain. Finally, there are transcranially delivered therapeutic modalities for transiently altering brain function such as transcranial magnetic stimulation (TMS). TMS works well on shallow anatomical brain structures and within relatively large volumes of tissue [22], [23]. If the early promise of neuromodulation by ultrasound bears fruit, our work and that of our colleagues will point the way for a new therapeutic neuromodulatory modality, one that alters brain function in smaller volumes of tissue at greater depth than current non-invasive technologies based on existing MRI-guided ultrasound devices [24].
